# Ptomains, Leucomains, Toxins and Antitoxins

**Published:** 1897-03

**Authors:** 


### Book Reviews.

Ptomains, Leucomains, Toxins and Antitoxins ; or, The Chemical Factors in the Causation of Disease. By Victor C. Vaughan, Ph. D., M. D., Professor of Hygiene and Physiological Chemistry, and Frederick G. Novy, M. D., Junior Professor of Hygiene and Physiological Chemistry in the University of Michigan. New (third) edition. In one 12mo volume of 603 pages. Philadelphia: Lea Brothers & Co. 1896.

The authors tell us that "it is now generally recognised that those diseases that cause the greatest mortality, and consequently are of the greatest importance, are in reality cases of poisoning. The poison may be possessed of life, and, therefore, under favorable conditions, capable of indefinite increase in quantity after its introduction into the body, or it may be dead when introduced or generated in the body. . . . . Pathogenic germs are living poisons, and every infectious disease is actually an intoxication. Not only are there chemical factors in the causation of disease, but specific chemical agents are now being employed in the prevention and cure of disease."

Every part of this book is intensely interesting, but especially impressive is the chapter on the history of the bacterial poisons. Beginning with a brief account of the investigations of Albert von Haller, this historical sketch is carried down to the present day.

The foods which are liable to contain bacterial poisons are enumerated and discussed, and a chapter is then devoted to the methods used in their examination.

Chapter V. treats of the General considerations of the relation of bacterial poisons to infectious diseases, and is naturally very interesting to the careful student.

Considerable space is then devoted to the consideration of the bacterial poisons in detail.

Chapter VIII., however, is one to which the progressive medical man would turn and read with great care and profit. It treats of immunity, antitoxins and serumtherapy. We are told by the authors that "the subject of immunity is one of the most interesting and at the same time one of the most perplexing problems with which the student of scientific medicine can busy himself. To render man immune to such deadly infectious diseases as tetanus, diphtheria, cholera, typhoid fever and tuberculosis is the task that now occupies the time and energy of many honest toilers in the laboratories of science." "Exposure to a given disease for successive generations is one of the factors in evolution of natural immunity." "We do not claim that this is an essential factor, nor will we attempt to estimate its relative importance, but will content ourselves with naming it as a factor." If the animal economy can become habituated to the use of such poison as morphine, cocaine or nicotine, why may it not become immune to the action of relatively large quantities of bacterial poisons? We must take into consideration, of course, the fact that immunity is often only a lessened susceptibility, and this point is thoroughly considered by the authors in the discussion of natural immunity. "It is enough to state here that natural immunity is essentially cellular, and that the formation of bactericides and antitoxins is dependent upon cellular activity of certain tissues of the invaded host."

Artificial or acquired immunity is then discussed at length, all of the latest investigations upon this subject being concisely and clearly presented to the reader.

The authors then take up the subject of antitoxins. "We know very little concerning these substances. They are believed to be proteid bodies, but this belief is not founded upon any positive knowledge. So far we can only recognise their presence by their effects." Behring finds that the diphtheria-antitoxin is not altered by peptic digestion. If this be true, it cannot be serum-albumin or globulin, but must be a nuclein, since this is the only proteid-like body in the blood that is not altered by peptic digestion."

"The relation of nuclein to immunity has been demonstrated recently by Vaughan, who has rendered rabbits immune to the diplococcus of pneumonia by previous treatments with nucleinic acid obtained from yeast. The injections of nuclein increase the number of polynuclear cells and thus strengthen the resistance of the body."

The preparation of antidiphtheritic serum is then briefly given. "This curative serum has now been used in the treatment of many thousands of children, and the following facts seem to be demonstrated: 1. The treatment is practically free from ill-effects. 2. The mortality has only been from one-half to one-fourth what it was during the year preceding the introduction of this agent."

Under the heading of The importance of bacterial products to the toxicologist, the authors say:

The presence in the cadaver of substances which give not only the general alkaloidal reactions, but respond to some of the tests which have hitherto been considered characteristic of individual vegetable alkaloids, must be of the greatest importance to toxicologists. The possibility of mistaking putrefactive for vegetable alkaloids should always be borne in mind by the chemist in making his medico-legal investigations.

While there is no doubt that this is a question of importance to the toxicologist, still, we are inclined to the belief that interference with the tests are many times, at least, exaggerated. There can be no doubt but that the humane man would give the accused the benefit of any doubt that might be produced by the interference with recognised characteristic tests, but in every instance these tests must be made with reagents which are known absolutely to be pure and strictly in accordance with those conditions which have been shown to produce required results. Then, again, the absolute purity of the solvent and the repeated purification of the alkaloid extracted are points of the greatest importance, which every chemist of training and experience knows. Under this heading of The importance of bacterial products to the toxicologist, the authors devote nearly ten pages to the consideration of "a morphine-like substance" and its interference with the tests for morphine when present in the body. After a careful consideration of all they have to say upon the subject, we are inclined to the verdict of the Scotch jury—not proven.

They tell us that the putrefaction which occurs in the dead body is anerobic. Has this been proven beyond a question of doubt? Is oxygen absolutely absent during the putrefaction of the dead body after burial? It appears to us that the results of the work of Mr. H. T. Smith, which were reported to the American Chemical Society at its midsummer meeting, rather point to the fact that putrefaction of dead bodies after burial does not interfere with the identification and detection of morphine.

The chemistry of the ptomains then follows, and here we have an excellent account of the individual ptomains, thus far isolated, their preparation, properties, chemical composition and physiological effects.

The leucomains are then considered from the standpoint of their history, preparation, properties, chemical composition and physiological effects.

Taken as a whole, this book is one of intense interest, and should be read and studied by every progressive student of scientific medicine.

The typography and general make-up of the book are excellent.

